# Ultrathin and Efficient Organic Photovoltaics with Enhanced Air Stability by Suppression of Zinc Element Diffusion

**DOI:** 10.1002/advs.202105288

**Published:** 2022-01-22

**Authors:** Sixing Xiong, Kenjiro Fukuda, Shinyoung Lee, Kyohei Nakano, Xinyun Dong, Tomoyuki Yokota, Keisuke Tajima, Yinhua Zhou, Takao Someya

**Affiliations:** ^1^ Center for Emergent Matter Science (CEMS) RIKEN 2‐1 Hirosawa Wako Saitama 351‐0198 Japan; ^2^ Wuhan National Laboratory for Optoelectronics Huazhong University of Science and Technology Wuhan 430074 China; ^3^ Thin‐Film Device Laboratory, RIKEN 2‐1 Hirosawa Wako Saitama 351‐0198 Japan; ^4^ Department of Electrical Engineering and Information Systems The University of Tokyo 7‐3‐1 Hongo, Bunkyo‐ku Tokyo 113‐8656 Japan

**Keywords:** air stability, high power‐per‐weight, mechanical deformability, ultrathin organic photovoltaics, zinc diffusion

## Abstract

Ultrathin (thickness less than 10 µm) organic photovoltaics (OPVs) can be applied to power soft robotics and wearable electronics. In addition to high power conversion efficiency, stability under various environmental stresses is crucial for the application of ultrathin OPVs. In this study, the authors realize highly air‐stable and ultrathin (≈3 µm) OPVs that possess high efficiency (15.8%) and an outstanding power‐per‐weight ratio of 33.8 W g^−1^. Dynamic secondary‐ion mass spectrometry is used to identify Zn diffusion from the electron transport layer zinc oxide (ZnO) to the interface of photoactive layer; this diffusion results in the degradation of the ultrathin OPVs in air. The suppression of the Zn diffusion by a chelating strategy results in stable ultrathin OPVs that maintain 89.6% of their initial efficiency after storage for 1574 h in air at room temperature under dark conditions and 92.4% of their initial efficiency after annealing for 172 h at 85 °C in air under dark conditions. The lightweight and stable OPVs also possess excellent deformability with 87.3% retention of the initial performance after 5000 cycles of a compressing–stretching test with 33% compression.

## Graphical abstract

The diffusion of zinc element from the electronic transporting layer (ZnO) to the interface of active layers in organic photovoltaics, resulting in performance deterioration, is confirmed by dynamic secondary‐ion mass spectrometry. By suppressing the zinc diffusion, ultrathin organic photovoltaics (≈3 µm) are obtained with exceptionally high power‐per‐weight ratio, sufficient air environmental stability, and appropriate mechanical durability.

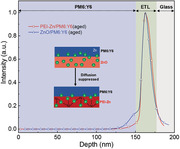

## 1 Introduction

Flexible organic photovoltaics (OPVs), being mechanically flexible and light weight, are regarded as one of the most promising independent portable power sources for driving wearable electronic devices.^[^ ^1^, ^2^ ^]^ Currently, the power conversion efficiency (PCE) of flexible OPVs is over 15%,^[^ ^3^, ^4^, ^5^, ^6^, ^7^, ^8^, ^9^ ^]^ which exceeds the typically assumed threshold efficiency for commercialization.^[^ ^10^ ^]^ In addition to their high efficiency, the storage, thermal, and light stabilities in air environments are further important performance indicators of flexible OPVs for practical applications.^[^ ^11^, ^12^ ^]^

To date, several strategies have been introduced to improve the stability of flexible OPVs. For example, the use of stable active layer materials is effective for improving their air storage stability.^[^ ^13^, ^14^ ^]^ Introducing a third component into the active layer was reported to improve the thermal stability of the mixed morphology.^[^ ^15^ ^]^ The application of an ultraviolet (UV) filter film was found to be an effective approach for improving the photostability of flexible OPVs.^[^ ^16^ ^]^ A recent study demonstrated that a flexible OPV with an outstanding PCE of 16.1%, based on a leaf‐like biomimetic design with efficient light capture and glossy surface, retained ≈75% of its initial efficiency for storage at 60 h in air.^[^ ^7^ ^]^

Recently, ultrathin OPVs with a total thickness of less than 10 µm have been developed to provide extreme mechanical compliance and deformability,^[^ ^17^, ^18^ ^]^ which is beneficial for integration with conformable wearable sensors or electronic skin (e‐skin).^[^ ^2^, ^19^, ^20^, ^21^, ^22^ ^]^ For example, an ultrathin OPV with a total thickness of 3 µm can be integrated with organic electrochemical transistors (OECTs) to realize self‐powered cardiac signal detection.^[^ ^2^ ^]^ A recent implementation of a ternary strategy that reduces crystallization and aggregation without decreasing the electron mobility has provided the best performance value of PCE of up to 15.5% and a high power‐per‐weight ratio of ≈32.1 W g^−1^ for ultrathin (≈3 µm) OPVs.^[^ ^23^ ^]^

However, highly efficient ultrathin OPVs (PCE > 15%) that can simultaneously achieve sufficient long‐term thermal (over 85 °C) and operational stabilities (under 1 sun) in air, have not been reported. Generally, preventing environmental effects is challenging for flexible OPVs, owing to the insufficient barrier of the flexible plastic substrate.^[^ ^1^ ^]^ For ultrathin OPVs, the effects of the environment can be even more severe because of the further reduction in the thickness of the plastic substrates.^[^ ^14^ ^]^ In addition to the intrinsic morphological changes in bulk heterojunction blends,^[^ ^24^, ^25^ ^]^ the changes in morphology, induced by the active layer/transporting layer interfaces, can also affect the cell performance over time.^[^ ^26^, ^27^ ^]^ This highlights the urgent need to understand the effects of internal changes in OPVs under environmental stresses and to prevent or minimize these changes. Some amine‐containing polymer electron transporting layers (ETLs), such as polyethylenimine (PEI), polyethylenimine ethoxylated (PEIE), and poly[(9,9‐bis(3*′*‐(N,N‐dimethylamino)propyl)‐2,7‐fluorene)‐alt‐2,7‐(9,9‐dioctylfluorene)] (PFN) have a tendency to react with the nonfullerene active layer, resulting in poor efficiency.^[^ ^28^, ^29^, ^30^ ^]^ Furthermore, polymer ETLs, such as PFN and poly[(9,9‐bis(3*′*‐((N,N‐dimethyl)‐nethylammonium)‐propyl)‐2,7‐fluorene)‐alt‐2,7‐(9,9‐dioctylfluorene)] (PFN‐Br), are required to be ultrathin, which makes the deposition difficult with the printing techniques.^[^ ^31^ ^]^ Sol‐gel zinc oxide (ZnO) ETL has the advantages of low cost, easy synthesis, printability, and good optical transmittance, which make zinc‐based ETLs an excellent candidate for ETL in OPVs.^[^ ^32^, ^33^ ^]^ Thus, it is important to focus on the stability of ZnO‐based OPVs.

In this work, using dynamic secondary‐ion mass spectrometry (D‐SIMS), the diffusion of zinc from the ETL (ZnO) into the interface of active layer was identified as the source of performance deterioration for the first time. We demonstrated that an ion‐chelating interface layer suppressing the zinc diffusion simultaneously enhances the performance, environmental stability, and mechanical durability of ultrathin flexible OPVs. The optimized ultrathin OPVs with a thickness of 3 µm exhibited the highest efficiency of 15.8% and power‐per‐weight ratio of 33.8 W g^−1^. To the best of our knowledge, this is the highest power‐per‐weight value among previously reported OPVs. Simultaneously, the ultrathin OPVs also exhibited appropriate storage, thermal, and light stability, maintaining 89.6% of the initial efficiency for 1574 h of storage in air under dark conditions at room temperature, 92.4% of the initial efficiency after 172 h annealing at 85 °C in air under dark conditions, and 77.1% of the initial efficiency after the operational stability test (maximum point power tracking [MPPT]) of 1500 min under irradiation of 1 sun in air. Furthermore, these efficient and stable OPVs exhibited appropriate mechanical durability and maintained an initial PCE of 87.3% even after 5000 compression–stretching cycles with 33% compression.

## 2 Results and Discussion

### 2.1 Device Structure and Stability

**Figure 1:**
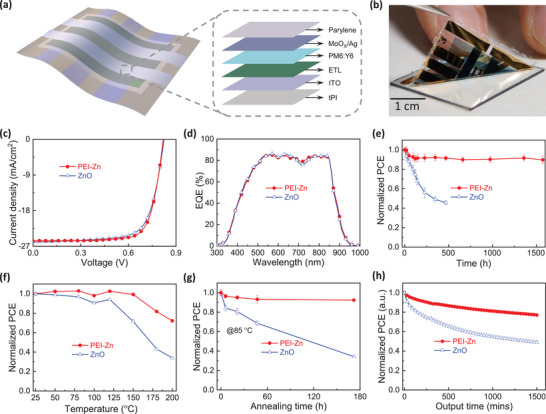
a) Structure of the ultrathin organic photovoltaics device. b) Photograph of the delamination process of the ultrathin OPV device from glass substrates. c) *J–V* characteristics of the ZnO‐ and PEI‐Zn‐based freestanding ultrathin photovoltaics with the best performance under illumination of 1 sun. The red solid circle and the blue open triangle indicate the PEI‐Zn and ZnO ETLs, respectively. d) EQE curves of freestanding solar cells based on ZnO and PEI‐Zn ETLs. Comparison of environmental stability in ambient air for freestanding ultrathin OPVs with ZnO (blue open triangle) and PEI‐Zn ETLs (red solid circle): e) storage stability at room temperature under dark conditions; and f) short‐term thermal stability in the temperature range of 25–200 °C under dark conditions (time steps of 5 min). g) Long‐term thermal stability at 85 °C under dark conditions. h) Operation stability based on MPPT under 1 sun illumination.

A schematic of the structure of the ultrathin OPVs is shown in **Figure** **1**a. An ultrathin OPV with an inverted structure of indium tin oxide (ITO) /ETL /active layer/MoO*_x_*/Ag was fabricated onto a 1.4‐µm‐thick transparent polyimide (tPI) substrate and encapsulated with a 1‐µm‐thick parylene layer. ZnO films synthesized by a sol‐gel method were used as the conventional ETL.^[^ ^34^, ^35^, ^36^ ^]^ Ethoxylated polyethyleneimine (PEIE) chelated with Zn^2+^ (PEI‐Zn) film was used as the ETL for the stable ultrathin OPVs.^[^ ^19^ ^]^ The active layer consisted of poly[(2,6‐(4,8‐bis(5‐(2‐ethylhexyl‐3‐fluoro)thiophen‐2‐yl)‐benzo[1,2‐b:4,5‐b′]dithiophene)alt‐(5,5‐(1′,3′‐di‐2‐thienyl‐5′,7′bis(2ethylhexyl)benzo[1′,2′‐c:4′,5′‐c′]dithiophene‐4,8‐dione)] (PM6) and 2,2′‐((2Z,2′Z)‐((12,13‐bis(2‐ethylhexyl)‐3,9‐diundecyl‐12,13‐dihydro[1,2,5]thiadiazolo[3,4‐e]thieno[2,″30″:4′,5′]thieno[2′,3′:4,5]pyrrolo[3,2‐g]thieno[2′,3′:4,5]thieno[3,2‐b]indole‐2,10‐diyl)bis(methanylylidene))bis(5,6‐difluoro‐3‐oxo‐2,3‐dihydro‐1H‐indene‐2,1‐diylidene))dimalononitrile (Y6). A blend of PM6:Y6 provides efficient charge transfer, separation, and transport; it is one of the most widely used active layers for high‐performance OPVs.^[^ ^37^, ^38^ ^]^ Figure S1, Supporting Information, shows the chemical structures of the PM6 and Y6 materials.^[^ ^39^ ^]^ MoO*_x_* and Ag were evaporated as the hole‐transporting layer and metal electrode, respectively. Finally, ultrathin OPVs with a total thickness of ≈3 µm were obtained. The detailed fabrication process is described in the experimental section (Supporting Information).

The ultrathin OPVs were delaminated from the supporting glass substrates after fabrication. A photograph of the peeling‐off procedure is shown in Figure 1b. Ultrathin OPVs with both the ZnO and the PEI‐Zn ETL did not show noticeable degradation in performance before and after the peeling process (as shown in Figure S2, Supporting Information), suggesting the sufficient repeatability of the ultrathin OPV fabrication. Figure 1c shows the current density–voltage (*J*–*V*) characteristics of the freestanding ultrathin OPVs with different ETLs. The freestanding OPVs with the ZnO ETL exhibited an open‐circuit voltage (*V*_OC_) of 0.83 V, a short‐circuit current density (*J*_SC_) of 25.40 mA cm^−2^, and a fill factor (FF) of 71.3%, thereby providing the highest PCE of 15.1% under the illumination of 1 sun. The ultrathin OPV with the PEI‐Zn ETL exhibited *V*_OC_ = 0.82 V, *J*_SC_ = 25.64 mA cm^−2^, FF = 75.2%, and the highest PCE of 15.8% under illumination of 1 sun. The OPVs with both the ZnO and the PEI‐Zn ETL exhibited negligible hysteresis (Figure S3, Supporting Information). The enhancement of the PCE with PEI‐Zn ETL can be attributed to the improvement of the FF, indicating that the PEI‐Zn ETL enhances the carrier collection efficiency compared to that of the ZnO ETL.^[^ ^40^, ^41^ ^]^ The statistics of parameters from 20 ultrathin freestanding solar cells are shown in **Table** **1**. The average PCE for the PEI‐Zn ETL was 15.2%, which was higher than that of the OPV with the ZnO ETL (14.5%). The distribution histograms of PCE are shown in Figure S4, Supporting Information. The narrower distribution of the efficiency in the ultrathin OPVs with PEI‐Zn ETL can be attributed to the better uniformity of the ETL film on the tPI substrate (Figure S5, Supporting Information). Both the ZnO‐ and the PEI‐Zn‐based solar cell fabricated on glass substrates exhibited comparable performances (Figure S6, Supporting Information). The current densities calculated from the external quantum efficiency (EQE) measurements (Figure 1d) were 24.91 and 24.96 mA cm^−2^ for the OPVs with ZnO and PEI‐Zn ETL, respectively, which was consistent with the *J–V* measurement (difference of ≈2%). A 4‐mm^2^ photomask was used to prevent the overestimation of the current density; however, no significant difference was found in the *J–V* curves before and after using the photomask (Figure S7, Supporting Information).

**Table 1:** Comparison of the performance of ultrathin OPVs based on different ETLs

ETL	*V*_OC_ [V]	*J*_SC_ [mA cm^−2^]	FF	PCE [%]
ZnO	0.823 ± 0.007	25.30 ± 0.70	0.70 ± 0.02	14.6 ± 0.5 (15.1) ^best^
PEI‐Zn	0.826 ± 0.005	25.70 ± 0.60	0.72 ± 0.02	15.2 ± 0.4 (15.8) ^best^

Statistics from 20 devices, the average efficiency is shown in the table.

The best efficiency is shown in parentheses.

Then, we studied the environmental stability of these freestanding ultrathin OPVs. When stored in air under dark conditions at room temperature, the performance of the devices with the ZnO ETL degraded to 45.6% of their initial efficiency after 468 h (Figure 1e). By contrast, the ultrathin OPVs with the PEI‐Zn ETL exhibited superior long‐term stability, maintaining 89.6% of their initial performance for 1574 h of storage. The variations in *V*_OC_, *J*_SC_, and FF as a function of time are plotted in Figure S8, Supporting Information. Both the *J*_SC_ and FF of the OPV with the ZnO ETL showed continuous deterioration and decreased to ≈70% of their initial value during 468 h of storage. By contrast, the OPVs with the PEI‐Zn ETL showed no change in *V*_OC_ and *J*_SC_, and the degradation was mainly due to that of FF.

We further investigated the thermal stability of the ultrathin OPVs in ambient air at temperatures in the range of 25–200 °C. The devices with PEI‐Zn ETL showed superior thermal stability compared to those with ZnO ETL (Figure 1f). The PCEs of the OPVs with PEI‐Zn ETL remained at 99.3% of their initial value even after heating at 150 °C and 72.2% of the initial PCE at 200 °C. The good stability at high temperatures is beneficial for the integration with wearable textiles.^[^ ^14^ ^]^ By contrast, the efficiency of the ZnO‐based OPVs degraded to 71.5% of their initial value after annealing at 150 °C for 5 min (the changes in *V*_OC_, *J*_SC_, and FF are shown in Figure S9, Supporting Information). A continuous high‐temperature (85 °C) stability test was also conducted (Figure 1g). The ultrathin OPVs with PEI‐Zn ETL retained 92.4% of their initial PCEs after 172 h, whereas the PCEs of the device with the ZnO ETL decreased to 34.3% of their initial values under the same conditions, indicating the superior thermal stability of the device with PEI‐Zn ETL. The changes in the *V*_OC_, *J*_SC_, and FF values are shown in Figure S10, Supporting Information. These results indicate that PEI‐Zn ETL significantly suppresses the degradation induced by thermal stress.

Furthermore, we compared the operational stability in air (maximum point power tracking [MPPT]) under the illumination of 1 sun. As shown in Figure 1h, the ultrathin OPVs based on PEI‐Zn ETL maintained 77.1% of their initial performance after 1500 min of continuous tracking, whereas the performance of the ZnO solar cells decreased to 49.1% of their initial value. The changes in the *V*_OC_, *J*_SC_, and FF values are shown in Figure S11, Supporting Information. The ultrathin OPVs with ZnO‐ETL showed a decrease in *J*_SC_ and FF, resulting in the deterioration of their PCE. By contrast, the device with PEI‐Zn ETL showed much more stable *V*_OC_, *J*_SC_, and FF values than those with the ZnO ETL. Furthermore, to simulate the real operation, we tested the maximum power point stability of the free‐standing OPVs under 1‐sun illumination at 50 °C. As shown in Figure S12, Supporting Information, the devices based on PEI‐Zn ETL maintained 74.8% of the initial efficiency for 610 min of continuous operation, whereas the efficiency of the device based on ZnO ETL decreased to 60.1% of the initial PCE for 170 min. We also studied the environmental stability of the rigid OPVs without an encapsulation layer (Figure S13, Supporting Information). The rigid devices based on PEI‐Zn ELT exhibited better stability than the ZnO‐based devices. These results indicate that the PEI‐Zn ETL significantly enhances the operational stability in air. The environmental stability of the ultrathin OPVs with PEI‐Zn ETL in air was superior to that of other flexible organic devices with high efficiency (>15%) (as summarized in Tables S1 and S2, Supporting Information).

### 2.2 Analysis of Morphology and Physical Mechanism

**Figure 2:**
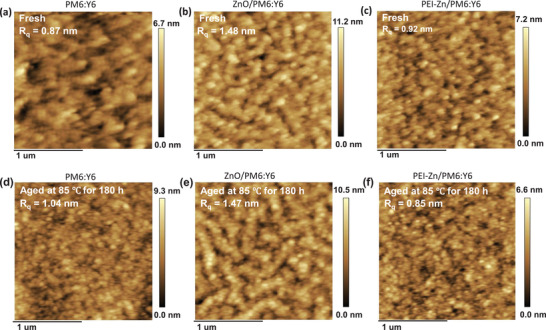
AFM height images of the as‐cast films of a) PM6:Y6, b) ZnO/PM6:Y6, and c) PEI‐Zn/ PM6:Y6. The images of d) PM6:Y6, e) ZnO/PM6:Y6, and f) PEI‐Zn/PM6:Y6 films after being aged in air for 180 h at 85 °C under dark conditions.

To study the degradation mechanism, accelerated aging testing was carried out at 85 °C in ambient air. Atomic force microscopy (AFM) was used with fresh and aged films to analyze the film morphology. **Figure** **2**a–c shows the AFM height images of the fresh PM6:Y6, ZnO/PM6:Y6, and PEI‐Zn/PM6:Y6 films on the glass substrate, respectively. All these fresh films had low root‐mean‐square roughness (*R*_q_) values. The AFM height images of the aged films are shown in Figure 2d–f. The *R*_q_ of PM6:Y6 increased from 0.82 to 1.04 nm after an aging process of 180 h. At the same time, the *R*_q_ value of ZnO/PM6:Y6 was not significantly affected by the aging treatment and the *R*_q_ of PEI‐Zn/PM6:Y6 decreased from 0.92 to 0.85 nm after aging. By contrast, a larger grain domain size was observed for the aged sample of the ZnO/PM6:Y6 film, whereas the aged PM6:Y6 and PEI‐Zn/PM6:Y6 films showed fine microstructures. The large domain size negatively affects the performance of OPVs.^[^ ^42^ ^]^ These results indicate that ZnO ETL accelerates the aggregation in the blend active layer.

**Figure 3:**
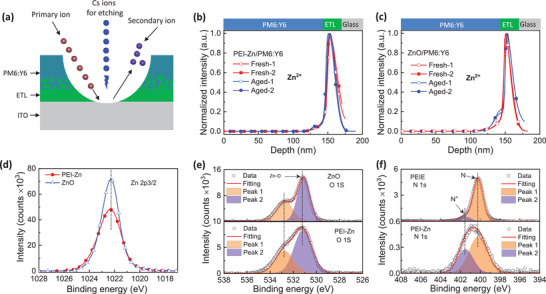
a) Schematic illustrations of the D‐SIMS measurement. Depth profiles of different samples from the pristine and aged samples under 85 °C 85% RH for 511 h: b) PEI‐Zn/PM6:Y6 and c) ZnO/PM6:Y6. Comparison of the d) Zn 2p and e) O 1s signals of the XPS results of ZnO and PEI‐Zn films. f) Comparison of the N 1s signals of the XPS results for PEIE and PEI‐Zn films.

To gain more insight into the mechanism resulting in the difference in the environmental stabilities, we employed D‐SIMS to observe the depth chemical profiles of the ETL and the PM6:Y6 active layer. The interface and depth profiles of the active layer and the ETL can be analyzed using D‐SIMS combined with etching technology. As shown in **Figure** **3**a, the primary ion source bombards the surface of the samples, and secondary ions are emitted. Etching of the film surface was performed using a Cs cluster ion beam. After dozens of etching cycles, the depth profiles of the films were analyzed.

We compared the D‐SIMS Zn ion signals of fresh and aged samples of glass/ETL/PM6:Y6. The aged samples were stored in an environmental chamber at a temperature of 85 °C with 85% relative humidity (RH) for 511 h. To eliminate the influence of the background, the Zn ion signal was normalized. Figure 3b shows the depth profiles of the Zn ion signal intensities in the aged and fresh samples of PEI‐Zn/PM6:Y6. Compared to the fresh samples, a slightly increased signal was observed in the aged samples at the interface between the PEI‐Zn and PM6:Y6 layers, indicating negligible Zn diffusion in the aged PEI‐Zn/PM6:Y6 samples. However, a significant enhancement of the Zn ion intensity can be observed at the interface between ZnO and PM6:Y6 in the aged ZnO/PM6:Y6 samples (as shown in Figure 3c). These results indicate that Zn diffuses from the ZnO layer to the interface of PM6:Y6 active layer; in contrast, the diffusion is suppressed when ZnO is replaced by PEI‐Zn. The absorption curves of the aged and fresh samples are shown in Figure S14, Supporting Information. The absorption of the aged ZnO/PM6:Y6 sample was blue‐shifted compared to that of the pristine sample. The changes in the absorption of the aged ZnO/PM6:Y6 compared to the fresh samples can be attributed to Zn diffusion.

To distinguish the properties of the ZnO and PEI‐Zn ETLs, X‐ray photoelectron spectroscopy (XPS) measurements were performed. As can be seen in Figure 3d, the peak binding energy of Zn 2p3/2 was 1022.3 eV for the pure ZnO and PEI‐Zn films, which can be attributed to the Zn—O bond.^[^ ^19^ ^]^ The Zn peak of the PEI‐Zn film was slightly broader than that of the ZnO film, indicating that the chemical environment of Zn in PEI‐Zn was less homogeneous than that in the ZnO film. In Figure 3e, the O 1s signals indicate the asymmetric peak fitted to the two peaks at 531.15 and 532.78 eV. The lower binding energy peak at 531.15 eV was assigned to the bonding of Zn—O, and the peak at 532.78 eV was assigned to the bonding of C═O in residual zinc acetates (Figure S15, Supporting Information). Figure 3f shows a comparison of the N 1s XPS signals of the PEIE and PEI‐Zn. The asymmetric N 1s signal was analyzed by fitting to the two peaks located at 400.2 and 401.5 eV. The peak with lower binding energy corresponded to non‐protonated nitrogen (N) and higher binding energy was assigned to protonated nitrogen (N^+^).^[^ ^28^, ^43^ ^]^ A small amount of protonated N^+^ in the PEIE samples originated from the H^+^ ions ionized by the solvent. The integrated area ratio of N^+^/N was found to be 0.1. However, the area ratio of N^+^/N in the PEI‐Zn film demonstrated a considerable enhancement. The N^+^/N ratio increased from 0.1 to 0.5, which resulted in the shifting of the binding energy of the N 1s XPS signal to blue. This indicates that a certain *n* amount of neutral N in PEIE transfer the electrons to Zn^2+^. By contrast, the ethanolamine evaporated under an ultra‐high thermal annealing temperature during the forming process of ZnO (as shown in Figure S16, Supporting Information). The residual zinc acetate precursor can be attributed to the insufficient conversion of precursors to ZnO, which typically requires a temperature of above 280 °C for full conversion.^[^ ^44^ ^]^ For the PEI‐Zn film, the PEIE materials bonded with Zn^2+^ in the residual precursor (Figure 3f). However, in the ZnO film, the ethanolamine evaporated during the annealing process (Figure S16, Supporting Information), indicating that the Zn^2+^ in the residual precursor could diffuse from the ZnO ETL into the PM6:Y6 active layer film without bonding. These results indicate that the interactions between the Zn ions from the zinc acetate and the amine group of the PEIE polymer during the PEI‐Zn forming process facilitate the suppression of Zn diffusion.

### 2.3 Power‐Per‐Weight Ratio and Mechanical Deformation of the Ultrathin OPVs

**Figure 4:**
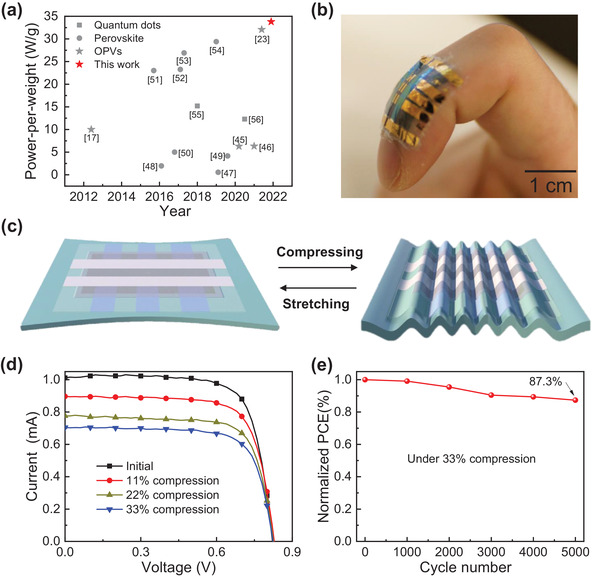
a) Comparison of power‐per‐weight ratios of our ultrathin OPV and reported results in the literature of solar cells. b) Photograph of the freestanding ultrathin OPVs attached on a bent finger. c) Schematic illustrations of pre‐stretching and compressing. d) *I–V* characteristics of the ultrathin device based on PEI‐Zn ETL under compression of 0%, 11%, 22%, and 33%. e) Normalized PCE of the PEI‐Zn‐based ultrathin OPV under cyclic compressing–stretching deformation with 33% compression.

Apart from the high efficiency and sufficient stability, other properties including power‐per‐weight ratio and mechanical deformation were also studied. The ultrathin OPVs with PEI‐Zn ETL exhibited a high efficiency of 15.8% and an area density of 4.67 g m^−2^, resulting in an excellent power‐per‐weight ratio of 33.8 W g^−1^. The power‐per‐weight calculation method is presented in the Supporting Information. To the best of our knowledge, this is the highest reported power‐per‐weight value for OPVs,^[^ ^17^, ^23^, ^45^, ^46^ ^]^ and in most cases even higher than those for other types of solar cells^[^ ^47^, ^48^, ^49^, ^50^, ^51^, ^52^, ^53^, ^54^, ^55^, ^56^ ^]^ (as shown in **Figure** **4**a). Using ultrathin substrates makes devices ultralight and simultaneously enables OPVs with excellent flexibility and deformability. As shown in Figure 4b, the ultrathin OPVs can be attached to a bending finger. Attaching ultrathin OPVs onto human skin or to the interface of soft robotics to harvest solar energy can significantly facilitate the development of self‐powered wearable electronics

To analyze the mechanical durability of the devices, we performed experiments using a compression test and a cyclic compression–stretching test. The ultrathin OPVs were transferred onto a pre‐stretched elastomer with 80% stretching. When the elastomer was released, the ultrathin OPV was compressed. A schematic diagram of the compression and stretching is shown in Figure 4c. The deformation is denoted as Δ*L*/*L*_0_, where *L*_0_ is the initial length when the elastomer is pre‐stretched, and Δ*L* is the change in length upon its release. Images of the pre‐stretching and compressing states are shown in Figure S17, Supporting Information. The current–voltage (*I*–*V*) characteristics of the ultrathin OPV under different compressions are shown in Figure 4d. The performance of the device based on PEI‐Zn ETL showed a continuous decrease in the compression range of 0–33%. An elastomer layer with a length of 20 mm was stretched to a length of 36 mm (*L*_0_). Then, the ultrathin OPVs were attached to the stretched elastomer. When the stretched elastomer was released to 24 mm, the ultrathin OPVs were compressed by 33%. Figure S18, Supporting Information, shows the variations in the parameters of *I*_SC_, *V*_OC_, and FF as a function of compression. The *V*_OC_ and FF values retained nearly 100% retention during compression, and only the *I*_SC_ value decreased due to the decrease in the effective irradiation area. A continuous compressing–stretching cycling test was performed (in the compression range of 0–33%) to analyze the mechanical durability. The ultrathin OPV based on the PEI‐Zn ETL showed 87.3% PCE retention after 5000 compression–stretching cycles with 33% compression (Figure 4e). The *J–V* characteristics and related parameters of *J*_SC_, *V*_OC_, and FF as a function of the compression cycling test are shown in Figure S19, Supporting Information. The degradation of the PCE was mainly due to the decrease in the *J*_SC_ and FF values. For comparison, the mechanical durability of ultrathin OPVs based on the ZnO ETL was also tested. The *I*–*V* characteristics of the ultrathin ZnO‐based device under the compression tests are shown in Figure S20a, Supporting Information. The ultrathin ZnO‐based device also exhibited appropriate mechanical durability with negligible changes in the *V*_OC_ and FF values during compression (Figure S20b, Supporting Information). The characteristics of the ultrathin OPVs based on the ZnO ETL after 5000 compressing–stretching cycles are shown in Figure S21a, Supporting Information. The PCE decreased to 84.4% of its initial value for the ZnO‐based ultrathin OPV after 5000 compression–stretching cycles with 33% compression. The related parameters of *J*_SC_, *V*_OC_, and FF as a function of the compression cycling test are shown in Figure S21b, Supporting Information. These results indicate that the ultrathin OPVs based on PEI‐Zn ETL can simultaneously deliver appropriate mechanical properties, high power‐per‐weight ratio, and sufficient air stability.

## 3 Conclusions

In summary, we demonstrated that zinc in the electron transport layer (ZnO) diffuses to the interface of active layer, resulting in performance degradation in air. By suppressing the zinc diffusion with a chelation strategy, the ultrathin OPVs exhibited appropriate storage, thermal, and operational stabilities in ambient air. These results suggest that the metallic oxide interface layer is also crucial for the stability of OPVs. The highly efficient and stable ultrathin OPVs exhibit a high power‐per‐weight ratio of 33.8 W g^−1^, enabling its potential application as a portable power source. The stable and ultrathin OPVs also demonstrated excellent deformability with 87.3% retention in performance after 5000 cycles of compression–stretching tests with a compression of 33%. The analysis of changes between the active and the metallic oxide interface layers can strongly support a better understanding of the degradation mechanism of OPVs.

## Conflict of Interest

The authors declare no conflict of interest.

## Author Contributions

S.X., K.F., Y.Z., and T.S. designed the study. S.X. and S. L. fabricated and optimized the ultrathin OPVs. S.X., K.F., K.N., and X.D. conducted the XPS measurements and analyzed the data. S.X. and K.F. conducted the SIMS measurements and analysis. S.X. contributed to the morphological analysis and mechanical tests. K.F., Y. Z., and T.S. coordinated the work. S.X., K. F., T. Y., K.T., and T.S. analyzed the data. S.X., K.F., Y.Z., and T.S. wrote the manuscript. All authors have revised and approved the manuscript.

## Supporting information

Supporting Information

## Data Availability

The data that support the findings of this study are available from the corresponding author upon reasonable request.
