# Supplementary material for: Ultrathin and Efficient Organic Photovoltaics with Enhanced Air Stability by Suppression of Zinc Element Diffusion
Source: Adv Sci (Weinh). 2022 Jan 22;9(8):2105288. doi: 10.1002/advs.202105288 (PMC8922108; doi:10.1002/advs.202105288)
Supplement: Supplementary file 1 — Supporting Information [file ADVS-9-2105288-s001.pdf]

ADVANCED SCIENCE
Open Access

Supporting Information

for *Adv. Sci.*, DOI 10.1002/advs.202105288

Ultrathin and Efficient Organic Photovoltaics with Enhanced Air Stability by Suppression of Zinc Element Diffusion

Sixing Xiong, Kenjiro Fukuda, Shinyoung Lee, Kyohei Nakano, Xinyun Dong, Tomoyuki Yokota, Keisuke Tajima, Yinhua Zhou* and Takao Someya**

WILEY-VCH

ADVANCED SCIENCE
Open Access

Supporting Information

for *Adv. Sci.,* DOI: 10.1002/advs.202105288

Ultrathin and Efficient Organic Photovoltaics with Enhanced Air Stability by Suppression of Zinc Element Diffusion

Sixing Xiong, Kenjiro Fukuda, Shinyoung Lee, Kyohei Nakano, Xinyun Dong, Tomoyuki Yokota, Keisuke Tajima, Yinhua Zhou,* and Takao Someya**

Supporting Information

Ultrathin and efficient organic photovoltaics with enhanced air stability by suppression of zinc element diffusion

Sixing Xiong, Kenjiro Fukuda, Shinyoung Lee, Kyohei Nakano, Xinyun Dong, Tomoyuki Yokota, Keisuke Tajima, Yinhua Zhou,* and Takao Someya**

Dr. Sixing Xiong, Dr. Kenjiro Fukuda, Dr. Shinyoung Lee, Dr. Kyohei Nakano, Prof. Keisuke Tajima, and Prof. Takao Someya
Center for Emergent Matter Science (CEMS), RIKEN, Wako, 351-0198 Saitama, Japan
E-mail: kenjiro.fukuda@riken.jp, takao.someya@riken.jp.

Dr. Sixing Xiong, Ms. Xinyun Dong, and Prof. Yinhua Zhou
Wuhan National Laboratory for Optoelectronics, Huazhong University of Science and Technology, Wuhan 430074, China
E-mail: yh_zhou@hust.edu.cn

Dr. Kenjiro Fukuda, and Prof. Takao Someya
Thin-Film Device Laboratory RIKEN, Wako, 351-0198 Saitama, Japan

Prof. Tomoyuki Yokota, and Prof. Takao Someya
Department of Electrical Engineering and Information Systems, The University of Tokyo, 113-8656 Tokyo, Japan

Experimental Section

Materials: The precursor (ECRIOS VICT-Cz) for the transparent polyimide (tPI) substrate was obtained from Mitsui Chemicals. The fluorinated polymers (Novec 1700 and 7100) were purchased from 3M Company. Zinc acetate dehydrates, ethanolamine, and 2-methoxyethanol were obtained from FUJIFILM Wako Pure Chemical Corporation. Ethoxylated polyethyleneimine (PEIE) was purchased from Sigma-Aldrich. PM6 and Y6 were purchased from 1-Materials. The chloroform solvent for the active layer solution was purchased from FUJIFILM Wako Pure Chemical Corporation. All materials were used as received without further purification.

Fabrication of ultrathin organic photovoltaics (OPVs): First, the glass substrates were treated with oxygen plasma for 10 min at 300 W. Then, the fluorinated polymer layer (Novec 1700:7100 = 1:8) was spin-coated (MS-B100, Miksa) on the glass at 4000 rpm for 1 min. Next, the fluorinated glass substrate was placed in an inert oven at 80 °C for 10 min. Before spin coating the tPI precursor, the glass/fluorinated polymer layer was treated with oxygen plasma for 5 s at 50 W. The precursor was spin-coated on the substrate at 3000 rpm for 1 min to form a film with a thickness of approximately 1.4 μm. The tPI film was cured by an imidization reaction at 250 °C for 8 h under an N_2 atmosphere (DN411I, Yamato Scientific). An indium tin oxide (ITO) transparent electrode of thickness 100 nm was deposited on the substrate using a sputtering machine (SIH-1010, ULVAC, Inc). The ITO electrode was patterned by photolithography, and then 3.5-nm-thick Cr and 100-nm-thick Au layers were

sequentially deposited onto the ITO electrode as contact pads. The tPI substrate with the ITO electrode was sequentially rinsed with acetone and 2-propanol. Subsequently, the substrates were blown dry using nitrogen flow. Before the deposition of ZnO, the substrate was treated with oxygen plasma at 300 W for 1 min (PC-300, SAMCO). The ZnO precursor solution was prepared by dissolving 109.8 mg zinc acetate dehydrate and 31.2 μL ethanolamine in 1 mL 2-methoxyethanol. The ZnO precursor solution was spin-coated on the tPI/ITO substrate at 3500 rpm for 45 s, followed by a thermal annealing at 180 °C for 30 min in air. The PEI-Zn precursor solution was prepared by dissolving 70 mg zinc acetate dehydrate in 1 wt% PEIE 2-methoxyethanol. To form the PEI-Zn film, the precursor solution was spin-coated at 3500 rpm for 45 s, and then thermally annealed at 180 °C for 30 min in air. PM6:Y6 (7 mg:9 mg) was dissolved in 1 mL chloroform:1-chloronaphthalene (1-CN) mixed solvent (99.5:0.5, volume ratio). The active layer solution was spin-coated at 3500 rpm for 45 s, and then annealed at 110 °C for 10 min in a glovebox. Then, a MoO_x hole-transporting layer (thickness of 7.5 nm) and an Ag electrode (thickness of 100 nm) were sequentially deposited (EX-200, ULVAC). The effective area of the solar cells was 4 mm^2. To connect the ultrathin OPVs, an external wiring on the polyimide substrates with Cr (3-nm-thick)/Au (100-nm-thick) was attached to the cathode and anode of the OPVs by electrically conductive adhesive tape (ECATT 9703, 3M Company). Finally, the devices were encapsulated by 1-μm-thick parylene (diX-SR, Daisan Kasei) evaporated by chemical vapor deposition (PDS 2010, KISCO Company).

Characterization of devices: The current density–voltage (J–V) characteristics of

ultrathin OPVs were measured using a Keithley 2400 Source Meter under an illumination of 1 sun using a solar simulator (AM 1.5 global spectrum calibrated using a silicon reference diode). For the stability test, all devices were stored in ambient air. For the maximum point power tracking (MPPT) test, the voltages at the maximum power point to the OSCs were continuously applied to the devices during the test. The voltage at the maximum power point was updated every 30 min based on the latest *J–V* characteristics measurement. The external quantum efficiency (EQE) measurements were performed with monochromatic light (SM-250F, Bunkoukeiki) calibrated using a silicon reference diode.

Characterization of the film: The absorbance of the films was characterized using an ultraviolet/visible/near infrared (UV-vis-NIR) spectrophotometer (V-780, JASCO) and the thicknesses were determined using a surface profiler (DEKTAK 6M, Bruker). Atomic force microscopy (AFM) was performed using a scanning probe microscope (SPM-9700HT, Shimadzu) in tapping mode. The dynamic secondary-ion mass spectrometry (D-SIMS) test (ADEPT-1010, ULVAC-PHI, Inc.) was performed by the TORAY Research Center, Inc. A photoelectron spectroscopy system (PHI5000 Versa Probe II, ULVAC-PHI Inc.) was used for the X-ray photoelectron spectroscopy (XPS) measurements.

Compression Test: The freestanding ultrathin OPVs were laminated on a pre-stretched elastomer (VHB Y-4905J, 3M). To control the amount of compression and stretching, a screw machine controlled by a program with pre-designed parameters was developed and manufactured in-house. The *J–V* curve was measured with a source

meter (2400 series, Keithley).

Calculation of the power-per-weight ratio: For the accurate calculation of the power-per-weight ratio of the fabricated ultrathin OPVs, the ITO, PEI-Zn, active layer, and MoO_x/Ag were completely formed on the transparent polyimide substrates (24 × 24 mm^2) without a pattern. To eliminate the nonuniformity at the edge, we cut the 24 × 24 mm^2 substrate to a size of 10 × 10 mm^2. The total weight of the three ultrathin OPVs (300 mm^2) was 1.4 mg (ATX224, Shimadzu).

Calculation of the power-per-weight ratio:

(a) The area density of the ultrathin OPVs is given by

$$\rho = \frac{m}{s}$$

where m is the mass quality and s is the area. Based on this equation, the quality density of our ultrathin OPVs was 4.67 g m^{-2}.

(b) The power density of the ultrathin OPVs can be calculated based on the power conversion efficiency (PCE) as

$$p = \mathrm{PCE} \cdot P_{in}$$

where P_{in} is the incident power (100 mW cm^{-2}, AM 1.5). Based on this equation, the highest power density was 158 W m^{-2}.

(c) The power-per-weight ratio of the ultrathin OPVs is given by the value of p/ρ. The power-per-weight ratio was 33.8 W g^{-1}.

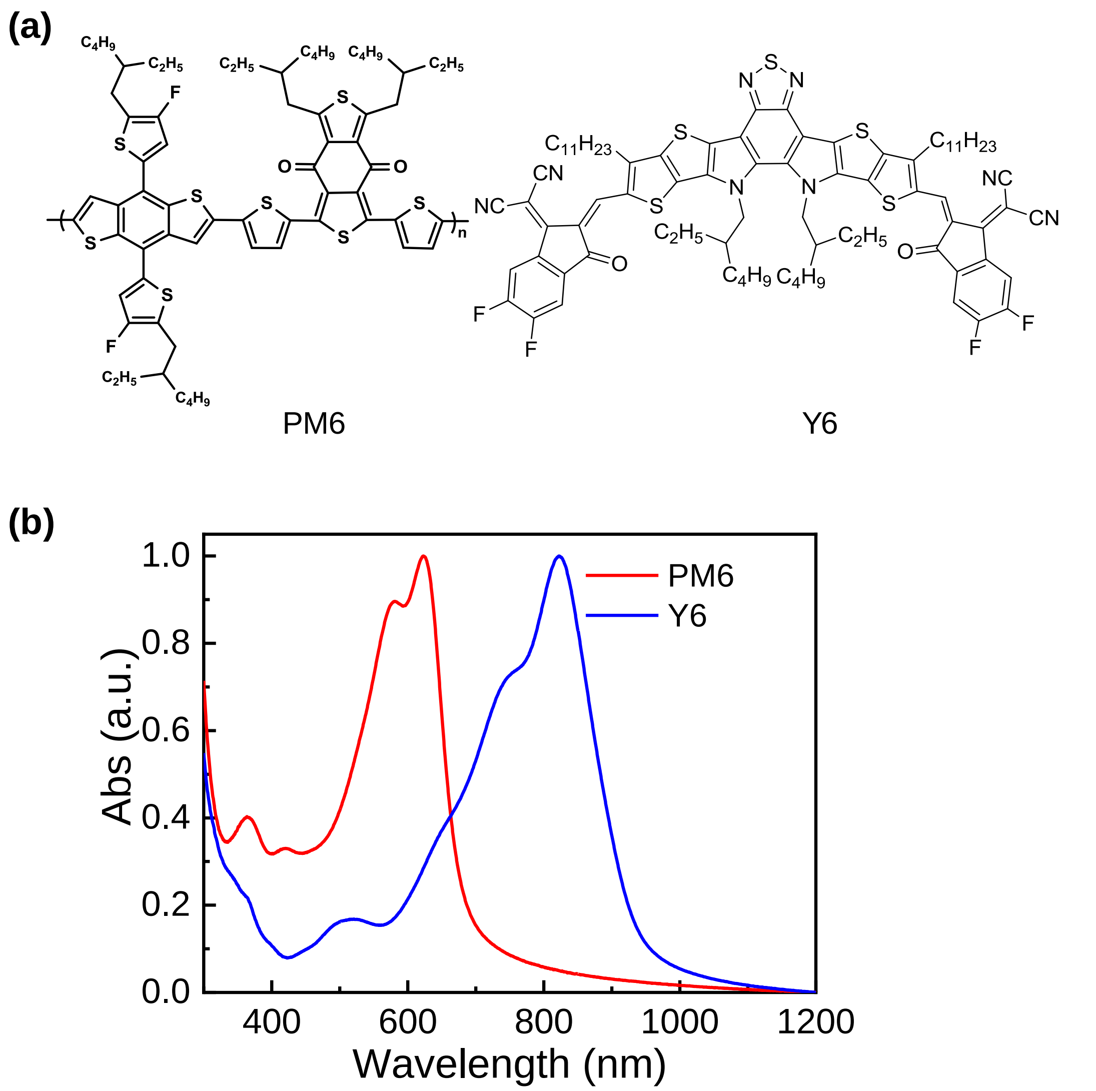

Figure S1 (a) Chemical structure of the PM6 donor polymer and Y6 acceptor. (b) Absorption curves of the PM6 and Y6 films.

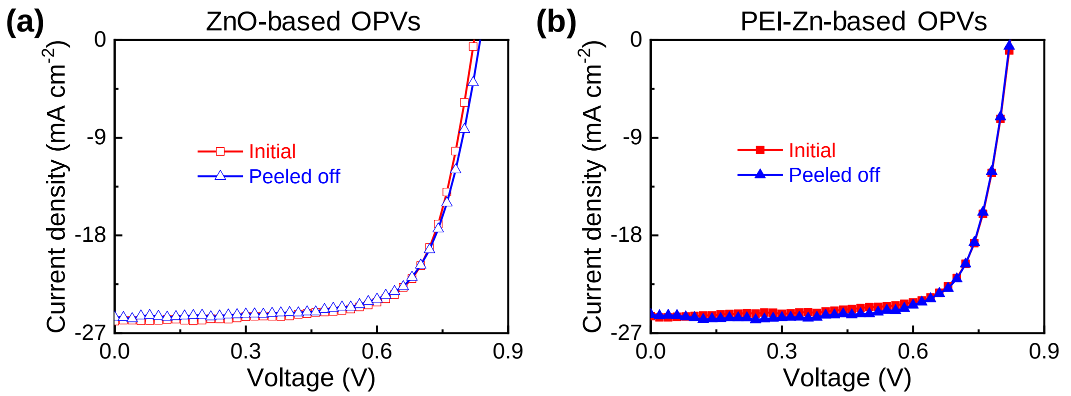

Figure S2 Comparison of the *J*–*V* curves of the OPVs before and after peeling from the glass substrates: (a) ZnO-based solar cells and (b) PEI-Zn-based solar cells. The initial performance parameters of the ZnO-based solar cells before peeling were as follows: V_{OC} = 0.82 V, J_{SC} = 25.8 mA cm^{-2}, FF = 70.6%, and PCE = 15.04%. After peeling off, the performance values of the freestanding OPVs with ZnO ETL were as follows: V_{OC} = 0.83 V, J_{SC} = 25.4 mA cm^{-2}, FF = 71.3%, and PCE = 15.07%. For the PEI-Zn solar cells, the initial performance parameters were V_{OC} = 0.82 V, J_{SC} = 25.4 mA cm^{-2}, FF = 74.7%, and PCE = 15.57%. After peeling off, the performance values changed to V_{OC} = 0.82 V, J_{SC} = 25.3 mA cm^{-2}, FF = 74.9%, and PCE = 15.55%. The performance before and after peeling off was nearly the same.

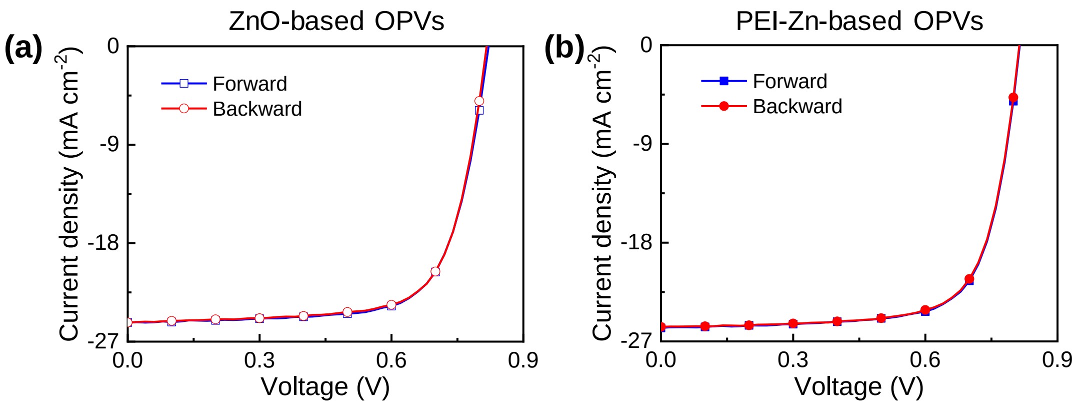

Figure S3 Hysteresis measurement of the ultrathin devices: (a) ZnO OPVs and (b) PEI-Zn OPVs. The forward scan and backward scan were performed from –1 to 1 V and from 1 to –1 V, respectively.

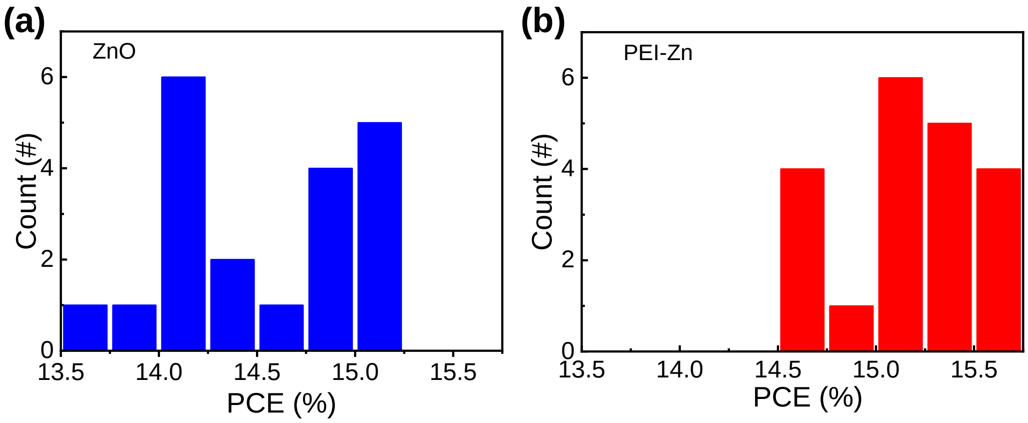

Figure S4 PCE statistics from 20 ultrathin freestanding solar cells. (a) Distribution histograms of the ZnO solar cells and (b) PCE of histograms for 20 PEI-Zn solar cells.

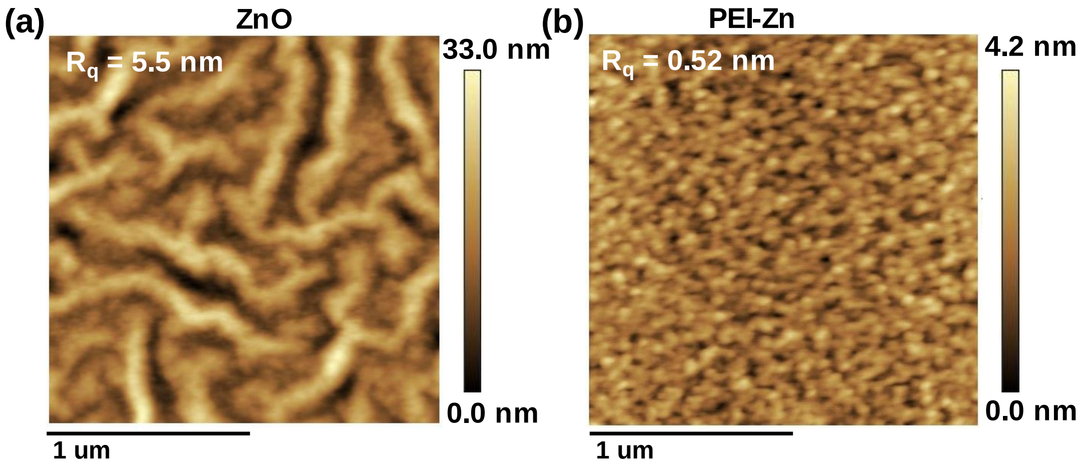

Figure S5 AFM images of (a) ZnO and (b) PEI-Zn films.

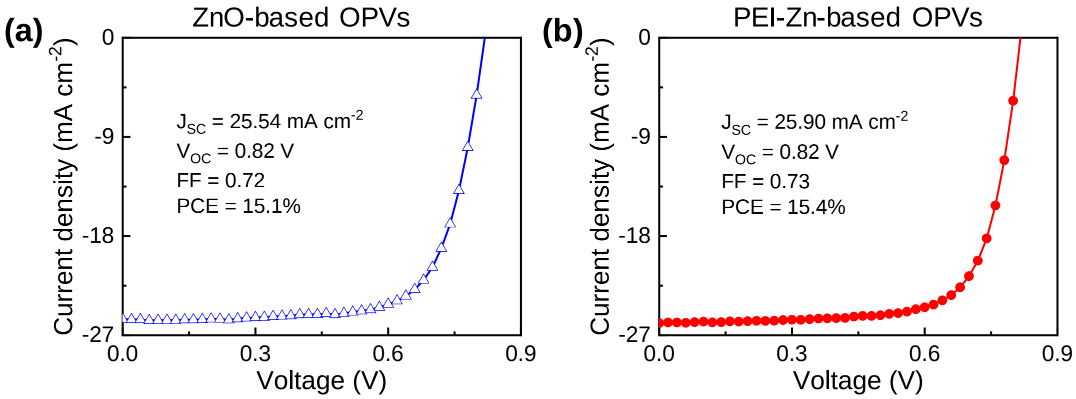

Figure S6 Performance of glass substrate devices based on different ETLs: (a) ZnO and (b) PEI-Zn.

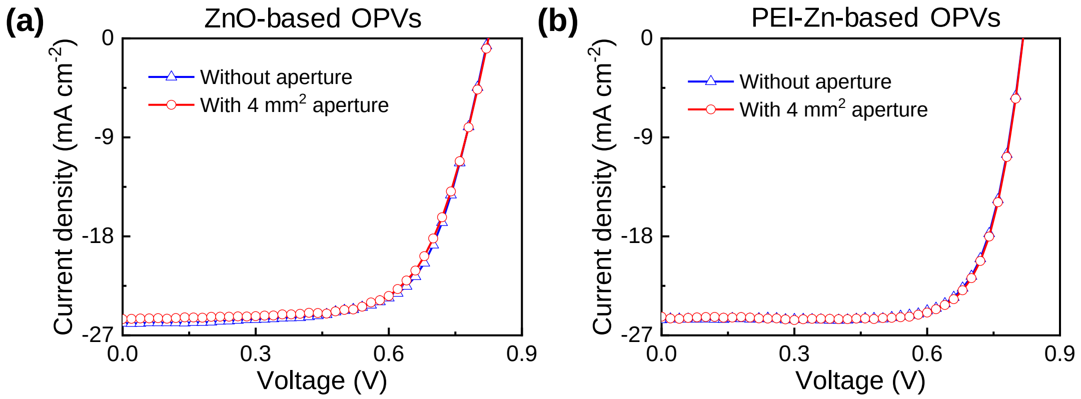

Figure S7 Performance of devices based on different ETLs with and without aperture: (a) ZnO and (b) PEI-Zn.

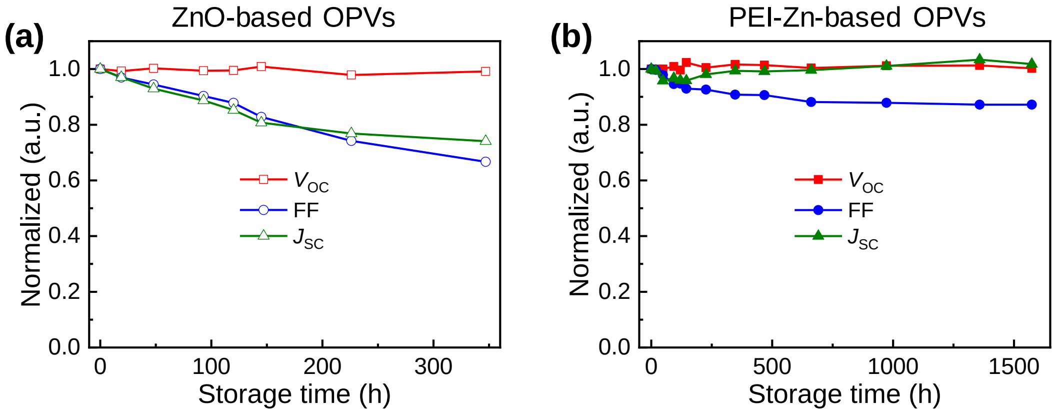

Figure S8 Evolution of V_{OC}, J_{SC}, and FF of different solar cells as a function of time when the devices were stored in air at 25 °C under dark conditions: (a) ZnO ETL and (b) PEI-Zn ETL solar cells.

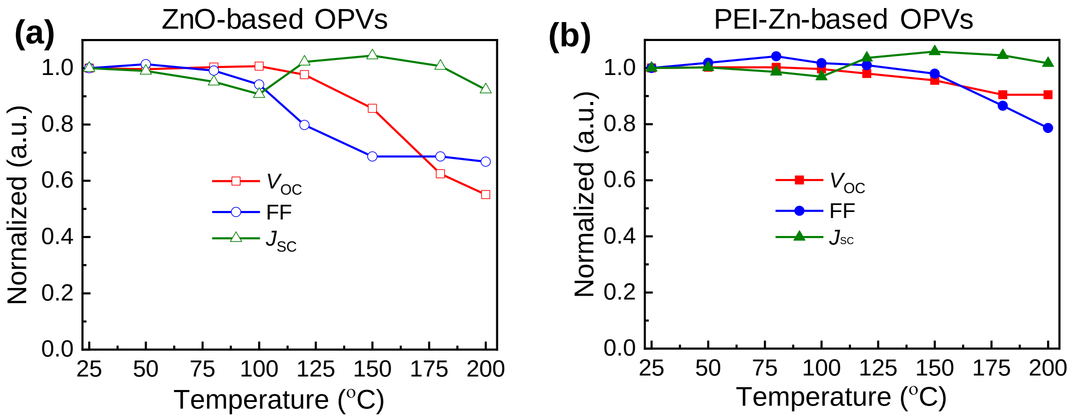

Figure S9 Evolution of V_{OC}, J_{SC}, and FF of different solar cells under different annealing temperatures in the range of 25–200 °C under dark conditions in air (in time steps of 5 min): (a) ZnO ETL and (b) PEI-Zn ETL solar cells.

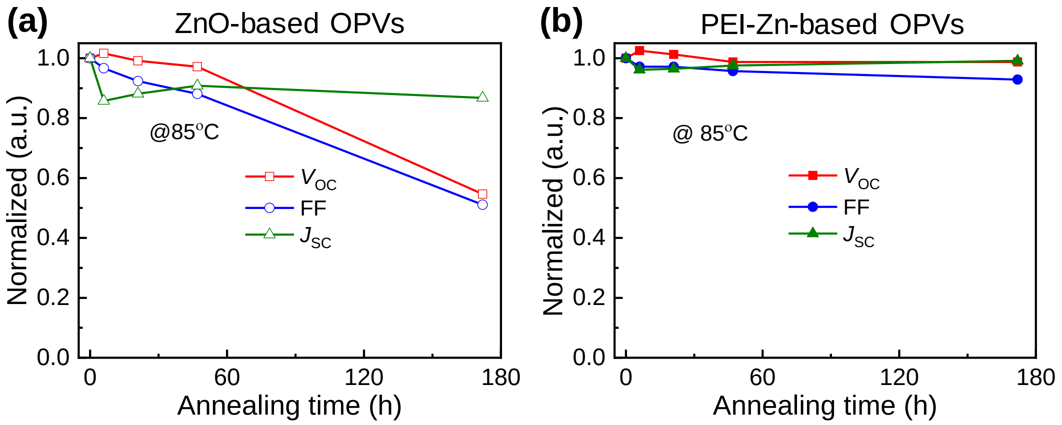

Figure S10 Evolution of V_{OC}, J_{SC}, and FF of different solar cells for long-term thermal stability under 85 °C in air: (a) ZnO ETL and (b) PEI-Zn ETL solar cells.

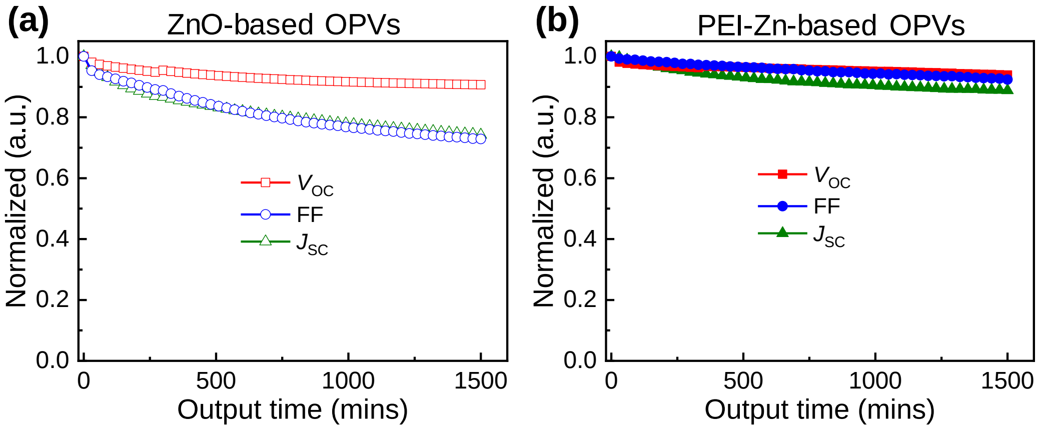

Figure S11 Evolution of V_{OC}, J_{SC}, and FF of different solar cells for MPPT measurement in air: (a) ZnO ETL and (b) PEI-Zn ETL solar cells.

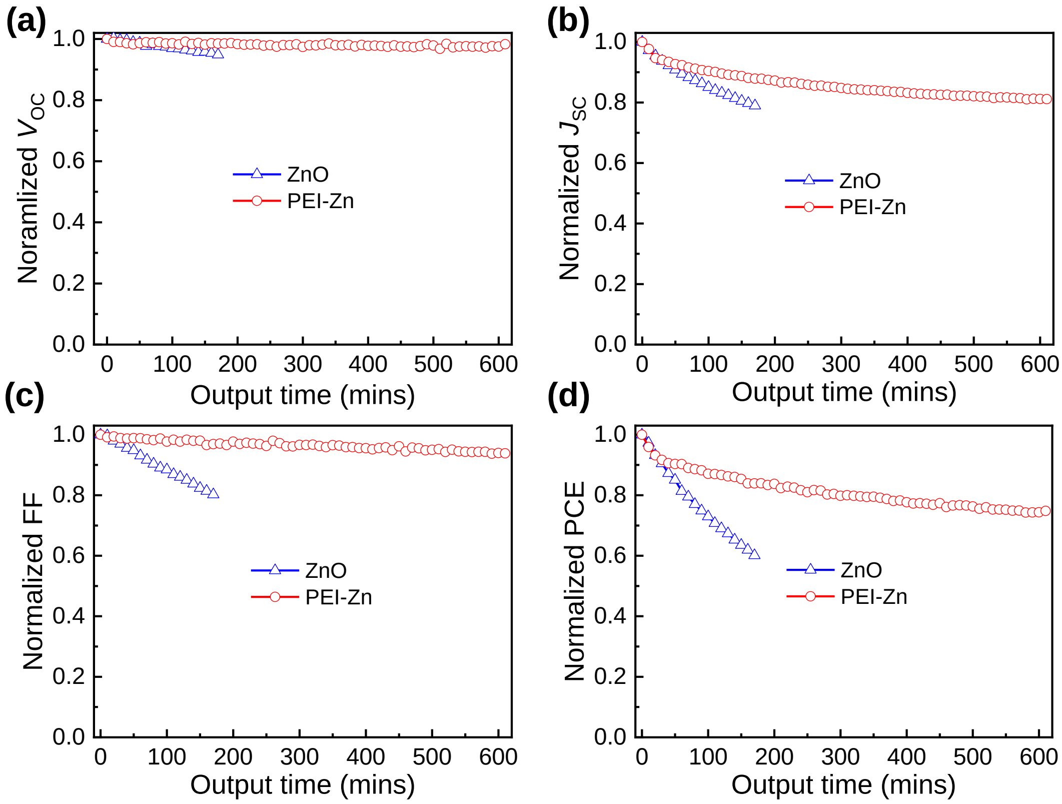

Figure S12 Evolution of V_{OC}, J_{SC}, FF, and PCE of different solar cells for MPPT measurement under continuous 50 °C annealing: (a) V_{OC}; (b) J_{SC}; (c) FF; and (d) PCE.

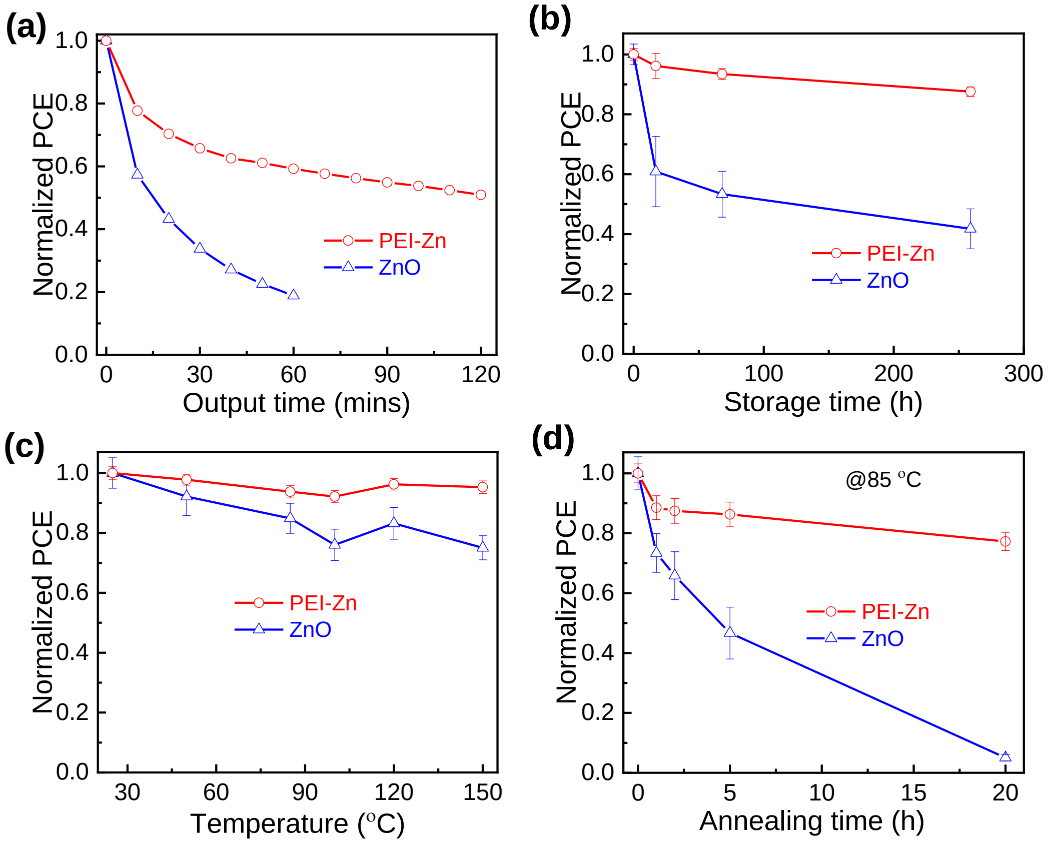

Figure S13 Comparison of environmental stability in ambient air of rigid OPVs without an encapsulation layer based on ZnO ETL (blue open triangle) and PEI-Zn ETL (red open circle): (a) Operation stability based on MPPT under 1 sun illumination. (b) Storage stability at room temperature under dark conditions; (c) Short-term thermal stability in the temperature range of 25–150 °C under dark conditions (time steps of 5 min). (d) Long-term thermal stability at 85 °C under dark conditions.

Table S1 Storage stability of representative flexible OPV devices (PCE > 15%) reported in the literature and in this work.

**Substrate** (Total thickness)	**ETL**	**HTL**	**Active layer**	**Environment**	**Time**	**Remaining**	**Ref.**
PET (Not mentioned)	ZnO (NPs) /PFN-Br	MoO_x	PM6:BTP-4F-12	In glovebox without encapsulation	Over 170 days	95.1%	S1
			PM6:Y6:$PC_{61}BM$			96.4%	
			PM6:Y6			95.3%	
PET (~3 µm)	PFNDI-Br	PEDOT:PSS	D18-Cl:Y6:$PC_{71}BM$	In glovebox without encapsulation	1000 h	90%	S2
PET (Not mentioned)	ZnO (NPs) /PFN-Br	MoO_x	PBDB-T:F-M/ PTB7-Th : $PC_{71}BM$:O6T-4F (Tandem devices)	In glovebox without encapsulation	Over 70 days	96%	S3
PET > 100 µm (glass encapsulation)	AZO	MoO_x	PBDB-T-2F:Y6	In air with glass encapsulation	144 h	61%	S4
PI (10 µm)	ZnO	MoO_x	PM6:N3:$PC_{71}BM$	In air without encapsulation	~60 h	~75%	S5
PI (~3 µm)	PEI-Zn	MoO_x	PM6:Y6	In air with parylene encapsulation	1574 h	89.6%	This work

Table S2 Thermal stability of representative flexible OPV devices (PCE > 15%) reported in the literature and in this work.

**Substrate** (Total thickness)	**ETL**	**HTL**	**Active layer**	**Environment**	**Time**	**Remaining**	**Ref.**
PET (Not mentioned)	PDINN	PEDOT:PSS	PM6:Y6	In glovebox at 85 °C	200 h	90.92%	S6
PI (~3 μm)	PEI-Zn	MoO_x	PM6:Y6	In air at 85 °C with parylene encapsulation	172 h	92.4%	This work

High-efficiency (> 15%) flexible solar cells with operational stability under 1 sun have rarely been documented. Here, we only listed the storage and thermal stabilities.

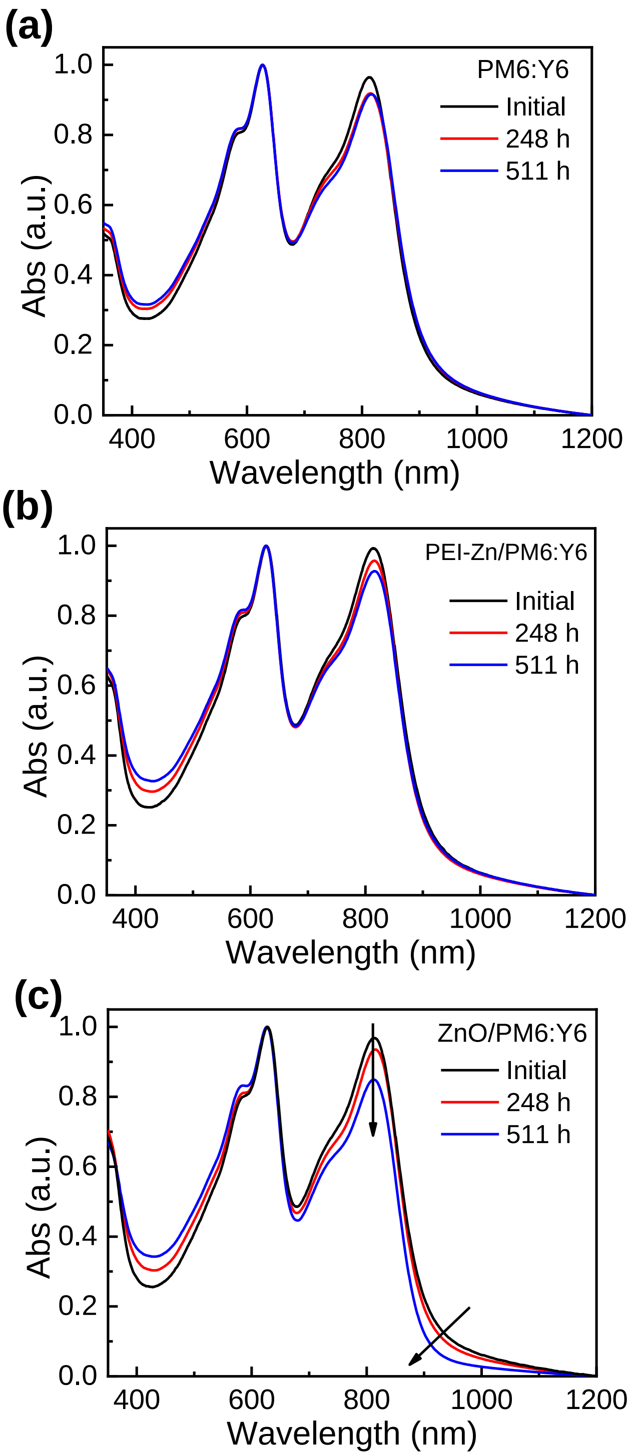

Figure S14 Absorption changes of different films aged under 85 °C 85% RH condition: (a) PM6:Y6 film; (b) ZnO/PM6:Y6 film; and (c) PEI-Zn/PM6:Y6 film.

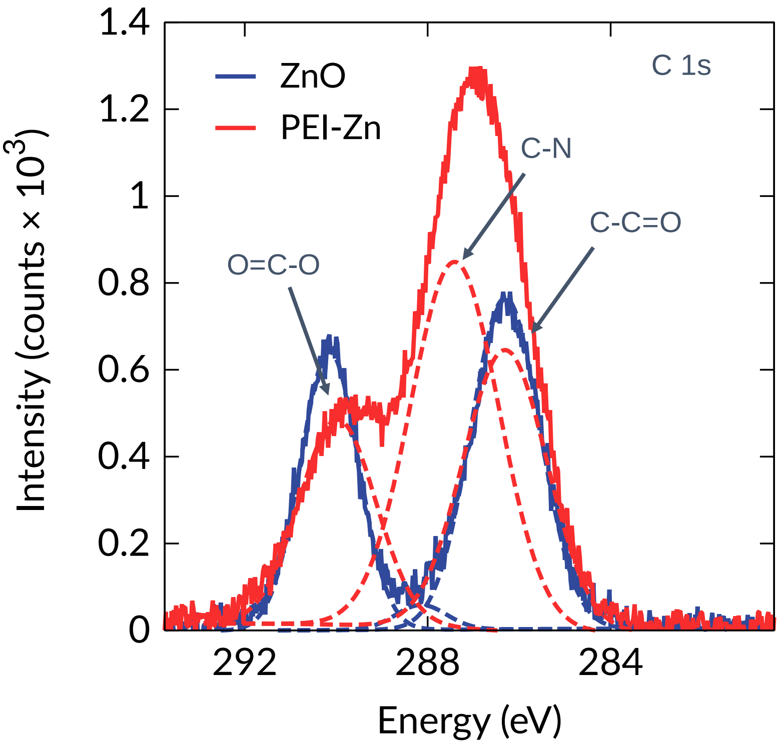

Figure S15 Comparison of C 1s signals of ZnO and PEI-Zn films in the XPS measurement.

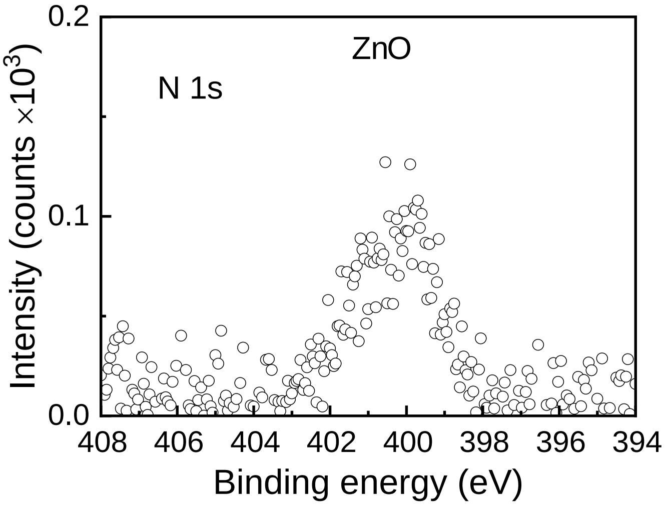

Figure S16 N 1s signal of the ZnO film in the XPS measurement.

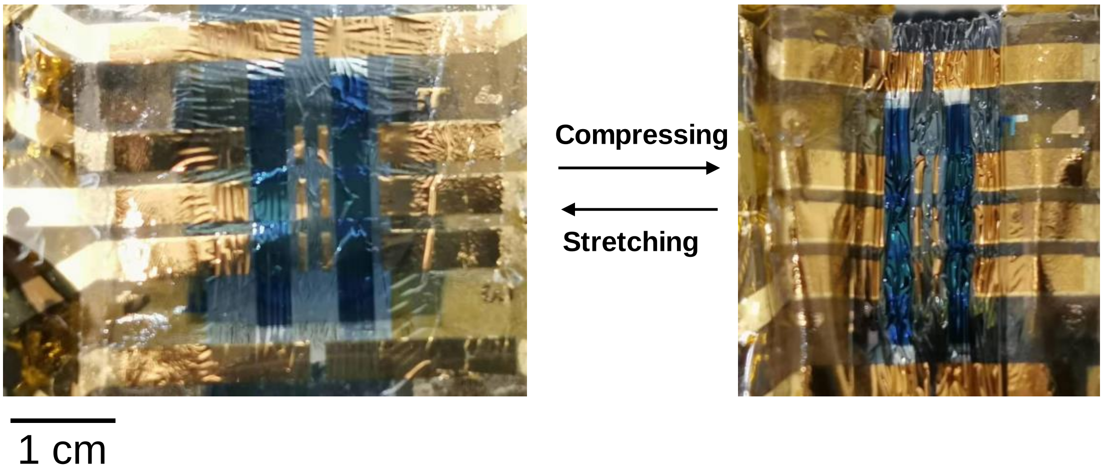

Figure S17 Photographs of the ultrathin OPVs during the stretching and compressing cycle measurement.

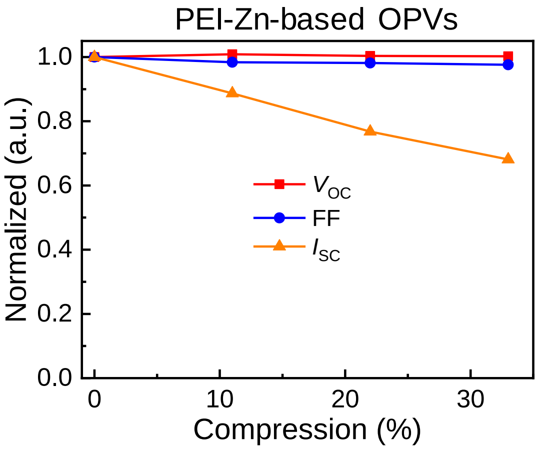

Figure S18 Evolution of V_{OC}, I_{SC}, and FF of the PEI-Zn ultrathin solar cells under different compressions.

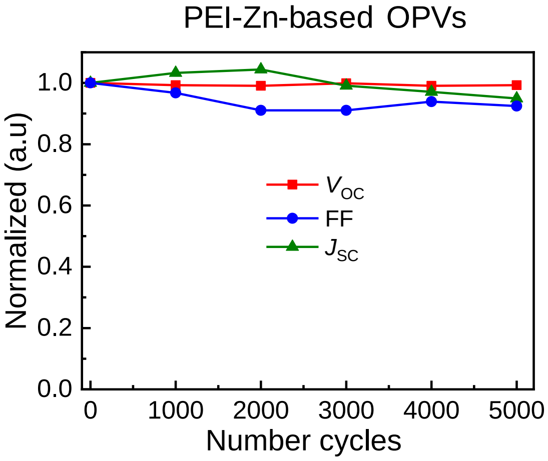

Figure S19 Evolution of V_{OC}, J_{SC}, and FF of the PEI-Zn-based ultrathin solar cells under different compressing–stretching cycles.

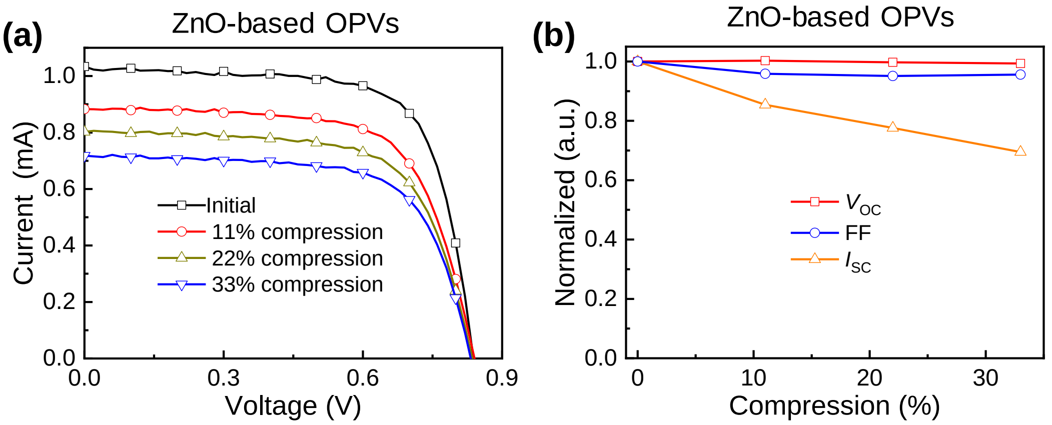

Figure S20 (a) Current–voltage (*I*–*V*) characteristics of the ultrathin OPVs based on ZnO ETL under compressions of 0%, 11%, 22%, and 33%. (b) Evolution of V_{OC}, J_{SC}, FF, and PCE of the ultrathin OPVs based on ZnO ETL under compressions of 0%, 11%, 22%, and 33%.

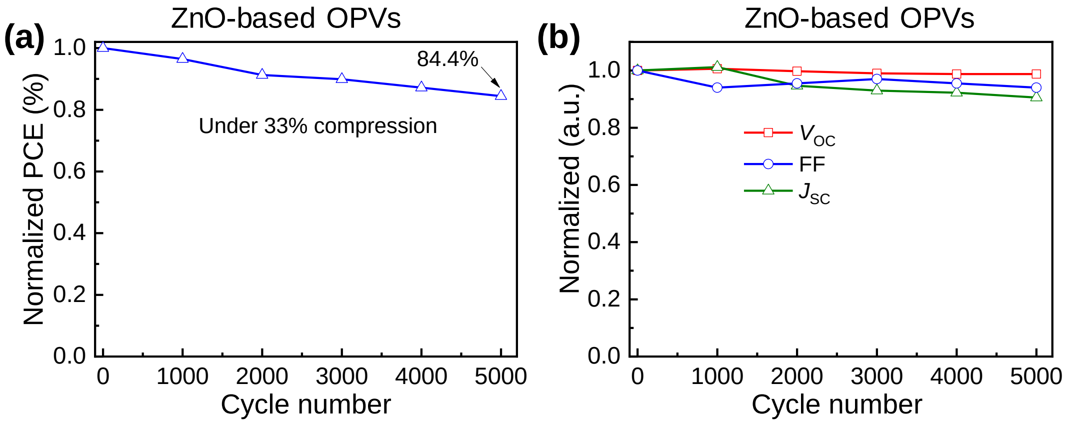

Figure S21 (a) Normalized PCE of the ultrathin OPV based on ZnO ETL under cyclic compressing–stretching deformation with 33% compression. (b) Evolution of V_{OC}, J_{SC}, and FF of the ultrathin OPVs based on ZnO ETL under cyclic compressing–stretching deformation with 33% compression.

References

S1. Y. Sun, L. Meng, X. Wan, Z. Guo, X. Ke, Z. Sun, K. Zhao, H. Zhang, C. Li and Y. Chen, *Adv. Funct. Mater.*, 2021, **31**, 2010000.

S2. W. Song, K. Yu, E. Zhou, L. Xie, L. Hong, J. Ge, J. Zhang, X. Zhang, R. Peng and Z. Ge, *Adv. Funct. Mater.*, 2021, **31**, 2102694.

S3. Y. Sun, M. Chang, L. Meng, X. Wan, H. Gao, Y. Zhang, K. Zhao, Z. Sun, C. Li, S. Liu, H. Wang, J. Liang and Y. Chen, *Nat. Electron.*, 2019, **2**, 513.

S4. X. Chen, G. Xu, G. Zeng, H. Gu, H. Chen, H. Xu, H. Yao, Y. Li, J. Hou and Y. Li, *Adv. Mater.*, 2020, **32**, 1908478.

S5. T.-Y. Qu, L.-J. Zuo, J.-D. Chen, X. Shi, T. Zhang, L. Li, K.-C. Shen, H. Ren, S. Wang, F.-M. Xie, Y.-Q. Li, A. K.-Y. Jen and J.-X. Tang, *Adv. Opt. Mater.*, 2020, **8**, 2000669.

S6. J. Wan, Y. Xia, J. Fang, Z. Zhang, B. Xu, J. Wang, L. Ai, W. Song, K. N. Hui, X. Fan and Y. Li, *Nano-Micro Lett.*, 2021, **13**, 44.
